# Health Effects of Natural Antioxidants

**DOI:** 10.3390/ijms241310792

**Published:** 2023-06-28

**Authors:** Mariarosaria Boccellino

**Affiliations:** Department of Precision Medicine, University of Campania “Luigi Vanvitelli”, 80138 Naples, Italy; mariarosaria.boccellino@unicampania.it

Free radicals are produced during metabolic processes in the human body and can lead to oxidative stress, cellular aging, and the development of various diseases. Antioxidants act as protective agents by neutralizing radicals [1,2]. While natural antioxidants present in food, such as vitamins, phenolic compounds, and carotenoids, are generally sufficient for normal needs, supplementation may be beneficial for specific health conditions. However, it is still unclear whether the protective effects are attributed to individual antioxidants or the unique combinations found in different foods. The bioavailability of natural antioxidants plays a crucial role in determining their health effects and their ability to exert biological activity in target tissues. Recent research suggests that natural antioxidants can play a protective role in cancer therapy by enhancing its effectiveness and reducing toxicity. 

This Special Issue, “Health Effects of Natural Antioxidants”, was established to highlight the latest discoveries regarding the molecular mechanisms underlying natural antioxidants, their bioavailability, and their potential therapeutic uses. It aimed to present the most recent advancements in the field, showcasing the significant impacts of natural antioxidants on health and their potential for therapeutic applications.

Mimura et al. conducted a study comparing the therapeutic potential of vitamin D3 (VitD) and paricalcitol (Pari) in controlling the development of experimental autoimmune encephalomyelitis (EAE), an animal model for multiple sclerosis (MS). EAE and MS are characterized by immune cell activation, the release of proinflammatory mediators, and demyelination in the central nervous system (CNS). While VitD has demonstrated immunomodulatory and antioxidant properties in controlling EAE, it can also trigger hypercalcemia. To address this issue, the authors sought to evaluate the efficacy of Pari, a non-hypercalcemic vitamin D analogue, in comparison to VitD in controlling EAE. Mice were injected with either VitD or Pari every other day after EAE induction. The researchers assessed various parameters including the recruitment of inflammatory cells, the mRNA expression of inflammatory markers, demyelination in the CNS, the production of proinflammatory cytokines, and inflammation in the gut. The results showed that VitD, but not Pari, exhibited down-modulatory effects by reducing the recruitment of inflammatory cells, suppressing the mRNA expression of inflammatory parameters, and attenuating demyelination in the CNS. Additionally, the EAE/VitD group showed lower production of proinflammatory cytokines by lymph-node-derived cells, reduced IL-17 production by gut explants, and decreased intestinal inflammation compared to the EAE untreated and Pari groups. Furthermore, the study investigated the differentiation of dendritic cells (DCs) in the presence of VitD or Pari. DCs differentiated in the presence of VitD displayed a more tolerogenic phenotype compared to those differentiated with Pari. Overall, the findings suggest that VitD, but not Pari, has the potential to be used as a preventive therapy to control the severity of MS. VitD demonstrated immunomodulatory effects, attenuated inflammatory responses, and protected against demyelination in the CNS. These results highlight the therapeutic potential of VitD in managing MS and provide insights into the immunological mechanisms underlying its beneficial effects [3].

In a study conducted by Tratnjek et al., the aim was to provide a detailed overview of the roles of vitamin A and retinoids in the chemoprevention and treatment of bladder cancer (BC). Bladder cancer (BC) is a prevalent malignancy associated with high recurrence rates and significant morbidity and mortality. The development of effective strategies for chemoprevention and treatment of BC is crucial. Epidemiological studies have suggested a potential link between adequate vitamin A intake and reduced BC risk. Retinoids, which are natural and synthetic derivatives of vitamin A, have gained considerable attention in cancer research due to their antioxidant properties and their ability to regulate cell growth, differentiation, and programmed cell death. Both in vivo and in vitro studies focusing on BC have demonstrated promising results regarding the potential use of retinoids in the prevention and treatment of this disease. However, the translation of these findings into clinical practice has been limited. This narrative review comprehensively discusses several key aspects related to vitamin A and retinoid metabolism, retinoic acid signaling, the pathobiology of BC, the epidemiological evidence supporting the role of dietary vitamin A in BC, mechanistic insights obtained from preclinical models, clinical trials involving retinoids, the limitations associated with retinoid use, innovative approaches for retinoid delivery, and the identification of components within retinoid signaling pathways that may serve as novel therapeutic targets. By providing an in-depth analysis of these topics, this review highlights the potential benefits and challenges associated with retinoid-based interventions in BC. An understanding of vitamin A metabolism and retinoid signaling pathways, as well as their complex involvement in the pathogenesis of BC, may offer valuable insights for the development of effective chemopreventive and therapeutic strategies. However, further research is needed to address the limitations and optimize the utilization of retinoids in clinical settings. The identification of novel delivery systems and the exploration of specific components within retinoid signaling pathways hold promise for enhancing the efficacy of retinoid-based therapies for BC [4].

Cotoraci et al. conducted a study with the objective of exploring the therapeutic potential of biologically active compounds and plant extracts in the treatment of various types of anemia. Anemia, characterized by low hemoglobin levels and reduced oxygen-carrying capacity, is a significant public health concern affecting individuals of all ages. While blood transfusion and oral iron supplements are commonly used treatments, they come with side effects and limitations that hinder their long-term use. This review aims to provide up-to-date information on the use of these compounds and plant extracts as alternative or adjunctive therapies for anemia. By examining the existing literature, the goal is to highlight their potential benefits, considering their natural origins and potential effectiveness in improving treatment outcomes. Ultimately, this review aims to contribute to our understanding of alternative therapeutic approaches for various types of anemia, expanding the available options beyond conventional treatments [5].

In a comprehensive review conducted by Jiménez-Cabrera et al., the primary objective was to examine the potential role of the Erythrina genus in managing inflammatory pain and its antioxidant properties. Oxidative stress and the generation of reactive oxygen species (ROS) have been implicated in the development and progression of various degenerative disorders accompanied by inflammation and pain. Despite the significant impact of oxidative stress on health, effective treatments for pain and inflammation remain a challenge. Multidisciplinary approaches are commonly employed in pain management, and traditional remedies, such as those derived from the Erythrina genus, have been utilized to alleviate pain and inflammation. The Erythrina genus is known to produce a diverse array of secondary metabolites, including flavanones, isoflavones, isoflavones, and pterocarpans, which possess antioxidant activity. Phenolic compounds, found in abundance in Erythrina plants, have demonstrated the ability to counteract pro-oxidants and inhibit key inflammatory signaling pathways, such as MAPK, AP1, and NFκB. While there is preclinical evidence supporting the use of Erythrina compounds for pain management, the precise pharmacological mechanisms underlying their effects are not yet fully understood. This review acknowledges the rapid advancements in drug-discovery-related disciplines but emphasizes that much of nature’s medicinal potential remains untapped. The antioxidant properties of Erythrina compounds may play crucial roles in mitigating oxidative stress and reducing inflammation-associated pain. Understanding the mechanisms of action and the pharmacological effects of these compounds could potentially lead to the development of novel therapeutic approaches for pain management. By deepening our understanding of the compounds derived from Erythrina plants and their mechanisms of action, we can potentially uncover new avenues for pain management and contribute to the advancement of drug discovery in this field [6].
